# Optical communication beyond orbital angular momentum

**DOI:** 10.1038/srep27674

**Published:** 2016-06-10

**Authors:** Abderrahmen Trichili, Carmelo Rosales-Guzmán, Angela Dudley, Bienvenu Ndagano, Amine Ben Salem, Mourad Zghal, Andrew Forbes

**Affiliations:** 1 University of Carthage, Engineering School of Communication of Tunis (Sup’Com), GreS’Com Laboratory, Ghazala Technopark, 2083, Ariana, Tunisia; 2 School of Physics, University of the Witwatersrand, Private Bag 3, Wits 2050, South Africa; 3 CSIR National Laser Centre, PO Box 395, Pretoria 0001, South Africa; 4 Institut Mines-Télécom/Télécom SudParis, 9 rue Charles Fourier, 91011 Evry, France

## Abstract

Mode division multiplexing (MDM) is mooted as a technology to address future bandwidth issues, and has been successfully demonstrated in free space using spatial modes with orbital angular momentum (OAM). To further increase the data transmission rate, more degrees of freedom are required to form a densely packed mode space. Here we move beyond OAM and demonstrate multiplexing and demultiplexing using both the radial and azimuthal degrees of freedom. We achieve this with a holographic approach that allows over 100 modes to be encoded on a single hologram, across a wide wavelength range, in a wavelength independent manner. Our results offer a new tool that will prove useful in realizing higher bit rates for next generation optical networks.

Since the beginning of the 21st century there has been a growing interest in increasing the capacity of telecommunication systems to eventually overcome our pending bandwidth crunch. Significant improvements in networks transmission capacity has been achieved through the use of polarization division multiplexing (PDM) and wavelength division multiplexing (WDM) techniques and also through implementing high order modulation formats^1^,^2^,^3^. However, it might not be possible to satisfy the exponential global capacity demand in the near future. One potential solution to eventually cope with bandwidth issues is space division multiplexing (SDM)^4^,^5^,^6^ and in particular the special case of mode division multiplexing (MDM), which was first suggested in the 1980s^7^. In MDM based communication systems, each spatial mode, from an orthogonal modal basis, can carry an independent data stream, thereby increasing the overall capacity by a factor equal to the number of modes used^8^. A particular mode basis for data communication is orbital angular momentum (OAM)^9^,^10^ which has become the mode of choice in many studies due to its topical nature and ease of detection with phase-only optical elements^11^,^12^. Indeed, OAM multiplexing implementation results have reported Tbit/s transmission capacity over both free space and optical fibers^13^,^14^. More recent reports have shown free space communication with a bit rate of 1.036 Pbit/s and a spectral efficiency of 112.6-bit/s/Hz using 26 OAM modes^15^. But, by taking into account the effects of atmospheric turbulence on the crosstalk and system bit error rate (BER) in an OAM multiplexed free space optics (FSO) link, experimental results have indicated that turbulence-induced signal fading will significantly deteriorate link performance and might cause link outage in the strong turbulence regime^16^,^17^,^18^. Recently, Zhao *et al*. claimed that OAM is outperformed by any conventional mode division multiplexing technique with a complete basis or conventional line of sight (LOS) multiple-input multiple-output (MIMO) systems^19^,^20^. Indeed, OAM is only a subspace of the full space of Laguerre Gaussian (LG) beams where modes have two degrees of freedom: an azimuthal index 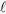 and a radial index *p*, the former responsible for the OAM. The addition of the radial degree of freedom certainly increases the bandwidth capacity, since for each value of 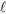 an infinite number of *p* values can be used to have access to many more information channels.

In this study, we demonstrate a new holographic tool to realise a communication link using a densely packed LG mode set incorporating both radial and azimuthal degrees of freedom. We show that it is possible to multiplex/demultiplex over 100 spatial modes on a single hologram, written to a spatial light modulator, in a manner that is independent of wavelength. Our subset of the LG modes were successfully used as information carriers over a free space link to illustrate the robustness of our technique. The information is recovered by simultaneously detecting all 100 modes employing a single hologram. Using this approach we are able to transmit several images with correlations higher than 98%. Although our scheme is a proof-of-concept, it provides a useful basis for increasing the capacity of future optical communication systems.

## Results

**Figure 1:**
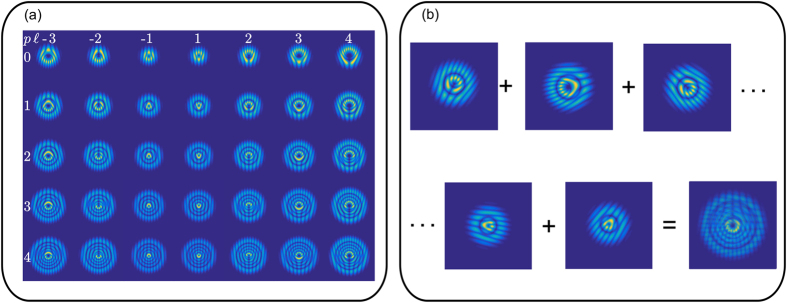
Complex amplitude modulation and spatial-multiplexing. (**a**) Holograms encoded via complex-amplitude modulation to generate different 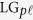 modes. (**b**) Holograms encoded with different carrier frequencies are superimposed into a single hologram to produce a spatial separation of all modes in the Fourier plane.

**Figure 2:**
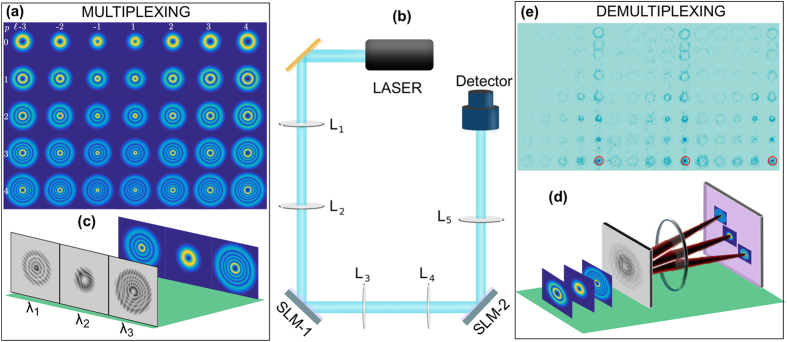
Schematic of our Multiplexing and Demultiplexing setup. (**a**) Intensity profiles of 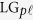 modes generated from combinations of *p* = {0, 1, 2, 3, 4} and 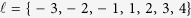. (**b**) Experimental setup: Three components of a multiline Ion-Argon laser, *λ*_1_ = 457 nm, *λ*_2_ = 488 nm and *λ*_3_ = 514 nm, are separated using a grating and sent to a Spatial Light Modulator (SLM-1). (**c**) The SLM is split into three independent screens, and addressed with holograms to produce the set of modes shown in (**a**). The information is propagated through free space and reconstructed in the second stage with a modal filter. (**d**) The modal filter consists of a superposition of all holograms encoded in SLM-2. (**e**) Each mode is identified in the far field using a CCD camera and a lens.

Consider a LG mode in cylindrical coordinates, at its waist plane (*z* = 0), described by:


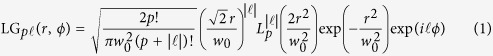


where *p* and 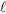 are the radial and azimuthal indices respectively, (*r*, *ϕ*) are the transverse coordinates, 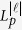 is the generalized Laguerre polynomial and *w*_0_ is a scalar parameter corresponding to the Gaussian (fundamental mode) radius. The mode size is a function of the indices and is given by 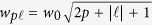. Such modes are shape invariant during propagation and are reduced to the special case of the Gaussian beam when 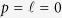. This full set of modes can be experimentally generated using complex-amplitude modulation. For this experiment we use the CGH type 3 as described in^21^ to generate a subset of 35 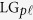 modes given by combination of *p* = {0, 1, 2, 3, 4} and 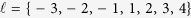. In this way, the amplitude and phase of the 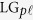 modes set (Eq. 1) can be encoded into phase-only digital holograms and displayed on phase-only SLMs to generate any 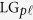 mode. Moreover, the holograms can be multiplexed into a single hologram to generate multiple modes simultaneously. Figure 1(a) shows the generated holograms to create the desired subset of 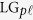 modes for this experiment. Their corresponding theoretical intensity profile can be seen in Fig. 2(a).

The 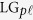 modes generated in this way were encoded using three different wavelengths onto a single hologram, in a wavelength independent manner, and sent through free space. At the receiver, we were able to identify with high fidelity any of the 105 encoded modes in a single real time measurement, using a wavelength independent multimode correlation filter on a single SLM^22^,^23^,^24^. This involves superimposing a series of single transmission functions *t*_*n*_(**r**), each multiplied with a unique carrier frequency **K**_*n*_ to produce a final transmission function *T*(**r**).


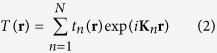


where *N* is the maximum number of multiplexed modes. In the Fourier plane the carrier frequencies **K**_*n*_ manifest as separate spatial coordinates as illustrated in Fig. 2(e). This approach allows multiple LG modes to be generated and detected simultaneously producing a high data transmission rate. The experimentally generated 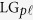 modes are used to encode and decode information in our multiplexing and demultiplexing scheme as shown in Fig. 2.

**Figure 3:**
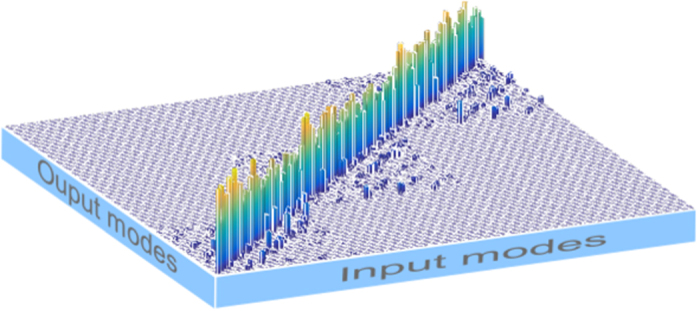
Cross Talk. For each input mode we measure the output cross talk for all hundred and five output modes. In all cases the input mode is detected with very high accuracy, higher than 98%.

To date only the azimuthal component, responsible for the OAM content of these mode, has been used for data transmission, ostensibly because the divergence is lowest for *p* = 0^9^,^19^. Here we demonstrate that the propagation dynamics, divergence being one example, is governed by the beam quality factor 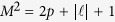
25 and that modes with the same index will propagate in an identical manner regardless of the radial component *p*. For example the modes LG_11_ and LG_03_ will experience the same diffraction since both has the same value *M*^2^ = 4. To show this, we encoded information in the set of 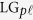 modes that incorporates both degrees of freedom, created as described before. Moreover, we multiplex the above mentioned subset of 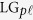 modes on three different wavelengths to increase our (de) encoding basis set from 35 to 105. All modes were generated using a single SLM (SLM-1 in Fig. 2(b)) and a wide range multi-line laser. The data are encoded using these mode set and transferred in free space. This information is recovered by projecting the propagated information onto a modal filter. The modal filter consists of multiplexed holograms displayed on a second SLM (SLM-2) and a CCD camera, capable of identifying with high accuracy any of the input modes (see experimental details). The intermodal crosstalk for the chosen modes, this is, the crosstalk between the input modes and the output modes was computed by measuring, for each input mode, the on-axis intensity of every output mode and normalized so that the total intensity adds up to unity (Fig. 3). As can be seen, the crosstalk between the different modes is very low and is independent of the *p* value. In a real scenario, where modes propagate over long distances, intermodal crosstalk would become an issue and the addition of compensating methods, as adaptive optics, would be needed.

**Figure 4:**
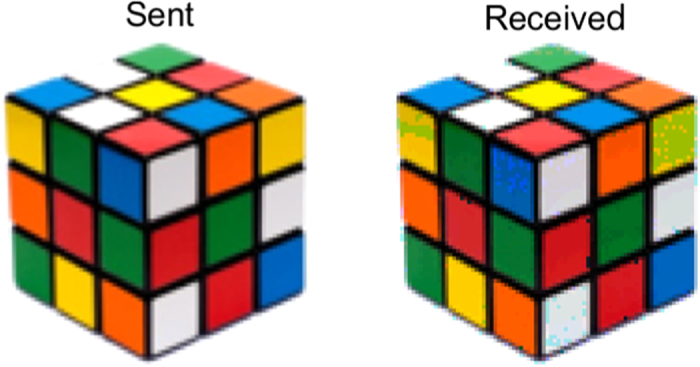
Example of sent and received images. A quantification of the similarity between sent and received images is done using 2D image correlation. The value of the correlation coefficient ranges from 0 for nonidentical images to 1 for identical images. The correlation coefficient for the above image is *c* = 0.96. Rubik’s Cube^®^ used by permission of Rubik’s Brand Ltd www.rubiks.com.

Figure 4 shows an example of an RGB image encoded, pixel by pixel as explained in the next section, and reconstructed in real time with a very high correlation coefficient (*c* = 0.96). The correlation coefficient is a dimensionless number that measures the similarity between two images, being 0 for nonidentical images and 1 for identical images.

### Encoding scheme

**Figure 5:**
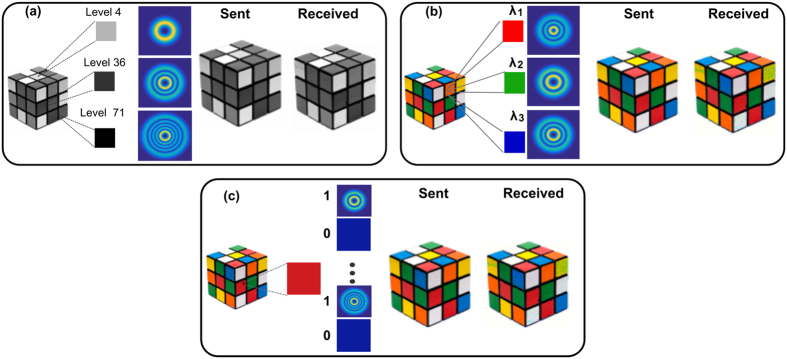
Encoding Configurations. (**a**) Single colour channel encoding, applied to gray-scale images. (**b**) RGB encoding, applied to colour images. (**c**) Multi-bit encoding, applied to both gray-scale and colour images. Rubik’s Cube^®^ used by permission of Rubik’s Brand Ltd www.rubiks.com.

The information encoding is performed in three different ways. In the first one, applied to grayscale images, we specifically assign a particular mode and a particular wavelength to the gray-level of each pixel forming the image. For example the mode LG_0–3_ generated with *λ*_1_ is assigned to the lowest gray-level and the mode LG_44_ generated with *λ*_3_ to the highest [see Fig. 5(a)]. In this approach we are able to reach 105 different levels of gray. In a second approach, applied to color images, each pixel is first decomposed into its three color components (red, blue and green). The level of saturation of each color is assigned to one of the 35 different spatial modes and to a specific wavelength *λ*_1_, *λ*_2_ or *λ*_3_ [see Fig. 5(b)]. In this approach only 35 levels of saturation can be reached with a total number of 105 generated modes. Finally, in the third we implement multi-bit encoding [see Fig. 5(c)]. In this scheme, 256 levels of contrast are achieved by multiplexing eight different modes on a single hologram. Each of the 256 possible permutations, of these 8 modes, representing a particular gray level. Upon arrival to the detector each permutation is uniquely identified and the information is decoded to its 8-bit form to reconstruct the image. This approach was extended to high contrast color images by using a particular wavelength for each primary color intensity, achieving a total rate of 24 bits per pixel. The reliability of our technique was further tested by transmitting different complex images containing all levels of saturation in each RGB component. The transmission error rate, defined as the ratio between the number of wrong pixels and the total number of transmitted pixels, is found very low and did not reach 1% in the case of gray-scale images and of 2% for RGB images. Here we only show the results for one image (Fig. 4), that clearly evinces the very high similitude between the original and recovered images.

## Discussion

Very recently it was pointed out that OAM multiplexing is not an optimal technique for free-space information encoding and that OAM itself does not increase the bandwidth of optical communication systems^19^,^20^. It has also been suggested that MDM, requires a complete mode set for a real bandwidth increment. Indeed, in all work to date only the azimuthal component of transverse modes, that gives rise to OAM, has been used in multiplexing schemes. Here we point out that the propagation dynamics (beam size, divergence, phase shift etc.) in free space are entirely governed by the beam quality factor, 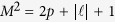
25, with analogous relations for fibre modes. The *M*^2^ can be viewed as a mode index: modes with the same index (e.g., *p* = 0, 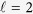 and *p* = 1, 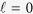) will propagate in an identical manner as they have the same space-bandwidth product (see supplementary information for some examples). It is clear that one mode set will be as good as any other (at least in terms of perturbation-free communication), provided that the elements are orthogonal and regardless of whether it carries OAM or not. To demonstrate this, we create a mixed radial and azimuthal mode set from the 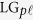 basis (with *p* = {0, 1, 2, 3, 4} and 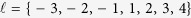) and use this to transfer information over free space. Moreover, by implementing MDM on different wavelengths, we demonstrate that it is possible to expand the overall transmission capacity by several orders of magnitude. The number of carrier channels would be given by the number of optical modes times the number of wavelengths. In our experiment we generated 35 optical modes and combined this with 3 different wavelengths, creating a basis set of 105 modes. These modes are used as information carriers in a proof-of-concept free space link, capable of transmitting and recovering information in real time with very high fidelity. Figure 4 is an example of the many images transmitted in our link. Each image is sent pixel by pixel, for this, the information of colour saturation of each pixel, is encoded using our mode set. Our encoding/decoding technique is key in the implementation of our optical link. Its simplicity linked to the versatility of SLMs, capable of operating in a wide range of the spectrum as well as with broad band sources, allowed us to generate customized digital hologram to encode and decode the information. Furthermore, the designed correlation filters are wavelength insensitive which allows the technique to operate in a large spectrum, compared to existing mode (de) multiplexers which are extremely wavelength sensitive, such as the photonic lantern. This approach can be extended to a wider range of wavelengths and to a higher number of modes, both limited by physical properties of the SLM as for example spatial resolution and wavelength operating range. The use of polarization could be potentially an additional degree of freedom and could possibly double the overall transmission capacity of the system. Even though here we have used our modes as information carriers, this experiment establishes the basis for this technique to be incorporated into standard communication systems. In this case each mode would represent a channel that can be modulated and detected with conventional technology.

To conclude, we have introduced a novel holographic technique that allows over 100 modes to be encoded/decoded on a single hologram, across a wide wavelength range, in a wavelength independent manner. This technique allowed us to incorporate the radial component of LG beams as another degree of freedom for mode division multiplexing. By combining both degrees of freedom, radial and azimuthal, with wavelength-division multiplexing, we are able to generate over 100 information channels using a single hologram. As a proof-of-concept, we implemented different encoding techniques to transmit information, with very high accuracy, in a free space link that employs conventional technology such as SLMs and CCD cameras. Our approach can be implemented in both, free space and optical fibres, facilitating studies towards high bit rate next generation networks. Additionally, our technique could also be extended to other type of orthogonal modes regardless of their OAM content, as for example Hermite-Gaussian beams.

## Methods

### Experimental details

The source, a continuum linearly-polarized Argon Ion laser (Laser Physics: 457–514 nm), is expanded and collimated by a telescope (*f*_1_ = 50 mm and *f*_2_ = 300 mm) to approximate a plane wave. Afterwards it is decomposed into its different wavelength components by means of a grating. Three of these components, *λ*_1_ = 457 nm, *λ*_2_ = 488 nm and *λ*_3_ = 514 nm propagating almost parallel to each other, are redirected to a HoloEye Pluto Spatial Light Modulator (SLM, 1080 × 1920 pixels) with a resolution of 8 *μ*m per pixel [see Fig. 2(b)]. The SLM is split into three independent screens, one for each beam, and controlled independently. Each third is addressed with a hologram representing a Laguerre-Gaussian mode 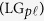, where *p* is the radial index and 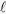 the azimuthal index [see Fig. 2(c)]. For this experiment we use 35 different modes [see Fig. 2(a)], generated by combinations of *p* = {0, 1, 2, 3, 4} and 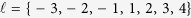. It should be stressed that the selection of the modes was made arbitrary and does not exclude any other combinations. These modes were encoded via complex amplitude modulations, a technique that allows for the generation of modes with purities higher than 0.98^26^. Additionally, we added blazed gratings to each hologram and spatially filtered the first diffraction order to generate wavelength independent holograms. Even though here we only used the first 35 LG modes, similar results would be obtained in using higher order modes and a larger set of modes, the limit is only imposed by the spatial resolution of the SLM.

The information decoding is performed using modal decomposition, for this, the beams are projected onto a second SLM using a 4*f* configuration system (*f*_3_ = 150 mm). This SLM is also split into three independent screens, each of which is addressed with a multiplexed hologram. This hologram consists of the complex conjugated of all 35 modes, encoded with different spatial carrier frequencies [see Fig. 2(d)]. To identify each mode, and therefore the graylevel of each pixel, we measured the on-axis intensity, of the projection, in the far field. For this we use a lens with focal length *f*_4_ = 200 mm and a CCD camera (Point Grey Flea3 Mono USB3 1280 × 960) in a 2*f* configuration system. In the detection plane (that of the camera), all 105 modes appear spatially separated, due to their unique carrier frequencies, in a rectangular configuration. In this way, an incoming mode can be unambiguously identified by detecting an on-axis high intensity [see Fig. 2(e)]. Even though, it is possible to get on-axis intensity for many other modes, the one that matches the incoming one, is always brighter. In our experiment, it is necessary to compensate for small spherical aberrations, this is done by digitally encoding a cylindrical lens on the second SLM which corrects for all modes. Notice that, since our modes, generated in SLM-1, were imaged onto SLM-2 for decoding, the effects as phase shift, beam size and divergence caused by propagation would be negligible and modes would seem to propagate identically. These effects would become stronger when propagating over long distances: modes in the same group would have identical properties but significant differences would exist between group.

## Additional Information

**How to cite this article**: Trichili, A. *et al*. Optical communication beyond orbital angular momentum. *Sci. Rep.* **6**, 27674; doi: 10.1038/srep27674 (2016).

## Supplementary Material

Supplementary Information
