# Supplementary material for: Optical communication beyond orbital angular momentum
Source: Sci Rep. 2016 Jun 10;6:27674. doi: 10.1038/srep27674 (PMC4901338; doi:10.1038/srep27674)
Supplement: Supplementary Information [file srep27674-s1.pdf]

Optical communication beyond orbital angular momentum

Abderrahmen Trichili[1], Carmelo Rosales-Guzmán[2,*], Angela Dudley[2,3], Bienvenu Ndagano[2], Amine Ben Salem[1], Mourad Zghal[1,4], and Andrew Forbes[2]

[1]University of Carthage, Engineering School of Communication of Tunis (Sup'Com), GreS'Com Laboratory, Ghazala Technopark, 2083, Ariana,Tunisia
[2]School of Physics, University of the Witwatersrand, Private Bag 3, Wits 2050, South Africa
[3]CSIR National Laser Centre, PO Box 395, Pretoria 0001, South Africa
[4]Institut Mines-Télécom/Télécom SudParis, 9 rue Charles Fourier, 91011 Evry, France
[*]carmelo.rosalesguzman@wits.ac.za

Supplementary information.

Normalization and Cross-Talk

Since the encoding technique employed for creating these holograms requires that the amplitude of the field be normalized to unity, energy conservation is violated in the generated modes. To compensate for this power scaling, a correction parameter is introduced for each transmission function. The correction parameter, α_n, is calculated as the ratio between the encoded optical field $\tilde{\Psi}_n(\mathbf{r})$ and the mode field $\Psi_n(\mathbf{r})$, where $\alpha_n = \max\{|\Psi_n(\mathbf{r})|\}^{-1}$ and $\alpha_n \in \Re^+$. In the case of the measurement procedure (i.e. performing the modal decomposition) the inner product can now be expressed as

$$\langle \tilde{\Psi}_n | \tilde{\Psi}_m \rangle = \langle \alpha_n \Psi_n | \alpha_m \Psi_m \rangle = \alpha_n \alpha_m \delta_{nm}, \tag{1}$$

where the correction coefficients are determined as a special case of equation (1)

$$\alpha_n^2 = \langle \tilde{\Psi}_n | \tilde{\Psi}_n \rangle \tag{2}$$

From the detection signal (i.e. the measured on-axis intensity, $I_n(\mathbf{r})$), the correction coefficients may be applied through the following relationship to normalise the signal to unit power:

$$I_n(\mathbf{r}) = \frac{\tilde{I}_n(\mathbf{r})}{\alpha_n^2} \tag{3}$$

The normalization is illustrated in Fig. S1 where the ratio between the energy of the generated mode and the energy of the demultiplexed signal is plotted for the various LG modes being used. The red data points contain the unnormalised measured signals illustrating a wider spread from unity (marked by the dotted line), while the blue data points contain the normalised signals which have a narrower spread.

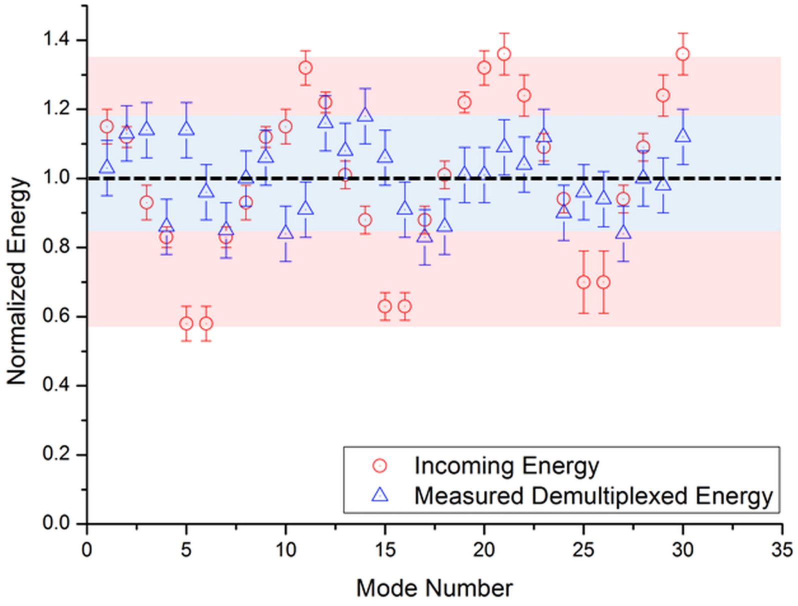

Figure S1. Normalization of detected signals. Plot of the the ratio of the energy in the generated modes with the energy in the detected signals as a function of the selected LG modes. The blue (red) data points contain the normalised (unnormalised) measured signals.

The effect of the aperture size in the detection plane when performing a modal decomposition on the incoming modes is also investigated. The selected LG modes (used as information carriers) were generated on SLM-1 and decomposed via an inner-product measurement at SLM-2 (as depicted in Fig. 2). The measurement results are presented in Fig. S2 which illustrate the (a) expected and measured cross-talk between neighbouring modes for a detection aperture size of (b) 24 μm (c) 9.6 μm and (d) 4.8 μm. In all cases [(b) - (d)] the strong diagonal and weak off-diagonal terms imply a highly accurate and precise measurement system which is unaffected by the size of the detection aperture.

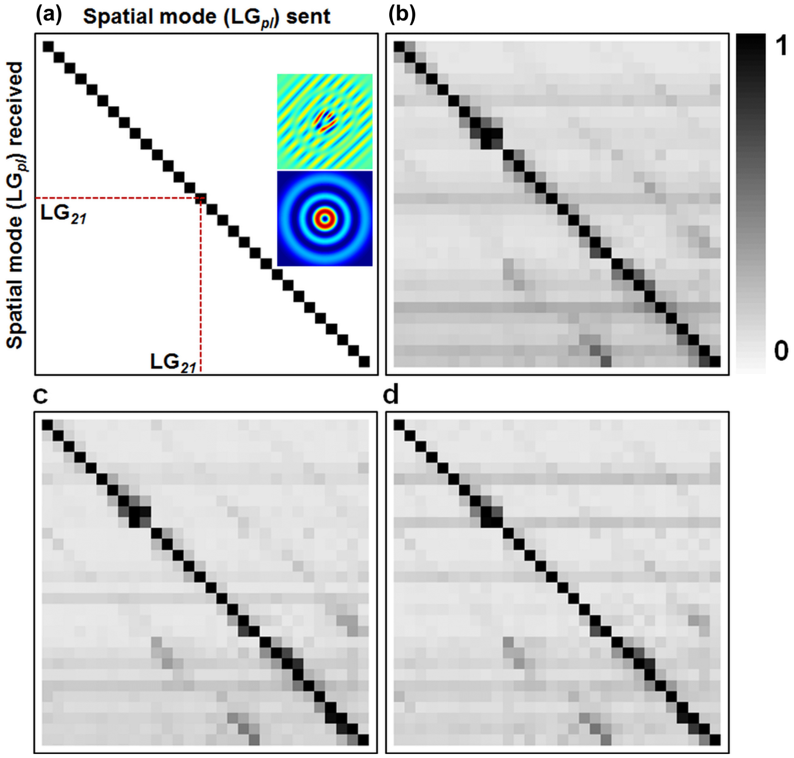

Figure S2. Effect of aperture size in the detection plane. Plot of the relative fractions of the intensity at each demultiplexed detector position for the selected LG modes. (a) The theoretical prediction and (b) - (d) measured cross-talk for aperture sizes of 24 μm, 9.6 μm and 4.8 μm, respectively.

Gray-Scale and Colour

The concept of assigning colour values present in a 2D image [as depicted in Fig. 5 (a) and (b)] was initially tested with a simple image such as those in Figs S3 (a) and (b). This test required ensuring that the detector positions were aligned correctly with the on-axis demultiplexed signals. Incorrect alignment would result in incorrect colour values being decoded. The initial test images contain either 30 different gray-levels [S3 (a)] or RGB-values [S3 (b)]. In both cases it is evident that the reconstructed images are in very good agreement with the sent images illustrating the correct alignment of the demultiplex signals with the static detector positions. The third image of Fig. S3 (b) contains a reconstructed image acquired four days after the system was initially aligned, illustrating its robustness and versatility.

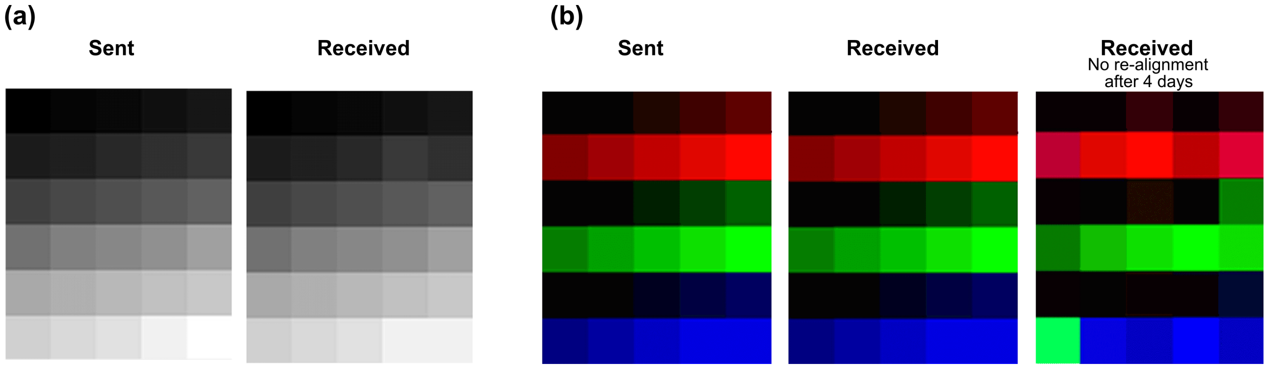

Figure S3. Gray-scale test. (a) The sent and received test image for verifying the success of the gray-scale encoding protocol. (b) The sent and received test image for verifying the success of the RGB encoding protocol.

8-Bit Encoding

In the gray-scale and RGB schemes the user is only concerned with detecting 1 signal out of a possible 35. However, the 8-bit scheme involves detecting 256 unique combinations of either no signal incremented in unit steps up to all possible 8 signals. Since the on-axis intensity is higher for a single signal as opposed to all 8 signals being present [demonstrated in Fig. S4 (a)], the user needs to carefully select the range of thresholds for the measured intensities. We investigate the impact the intensity threshold has on our encoding scheme and our findings are presented in Fig. S4 (b). The red border marks the sent image and the green border the successfully reconstructed image obtained at a suitable threshold. The images in between denote the reconstructed images when the threshold was initially set too low illustrating either extreme or mild cross-talk with neighbouring gray-values. Most of the cross-talk occurs with the white colour-value because when the threshold is set too low, the detectors detect noise - often resulting in 8 signals being detected which is the trademark of the white colour-value [Fig. S4(a)].

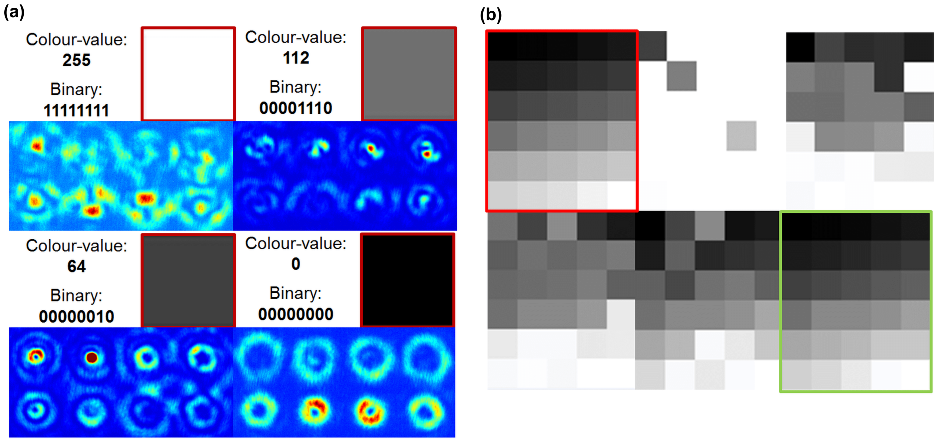

Figure S4. Threshold test for 8-bit encoding scheme. (a) Four selected gray-scales with their corresponding binary values and CCD images depicting the signals recorded at the detection plane. (b) The sent (red) and reconstructed gray-scale images for varying threshold values. Green marks successful reconstruction.

Beam quality factor

Through the beam quality factor, $M^2 = 2p + |\ell| + 1$ we can identify the LG modes that will propagate in an identical manner. Figure S5 shows the cross-talk table for each of the wavelengths we used in our experiment, in each table, we highlight some examples of the beams characterized by the same M^2 factor. Fig. S5 (a) corresponds to $\lambda_1 = 457$ nm, here the lines in red show four modes LG_{0-3}, LG_{11}, LG_{03} and LG_{1-1} (see insets) that share the mode index, $M^2 = 4$. In Fig. S5 (b) we highlight in green the modes corresponding to $\lambda_2 = 488$ and $M^2 = 6$ which are LG_{1-3}, LG_{2-1}, LG_{13}, and LG_{21}. As a final example, Fig. S5 (c) shows the cross-talk table for λ_3=514 nm, highlighted in orange the modes with $M^2 = 8$. This is, LG_{3-1}, LG_{23}, LG_{31} and LG_{2-3}.

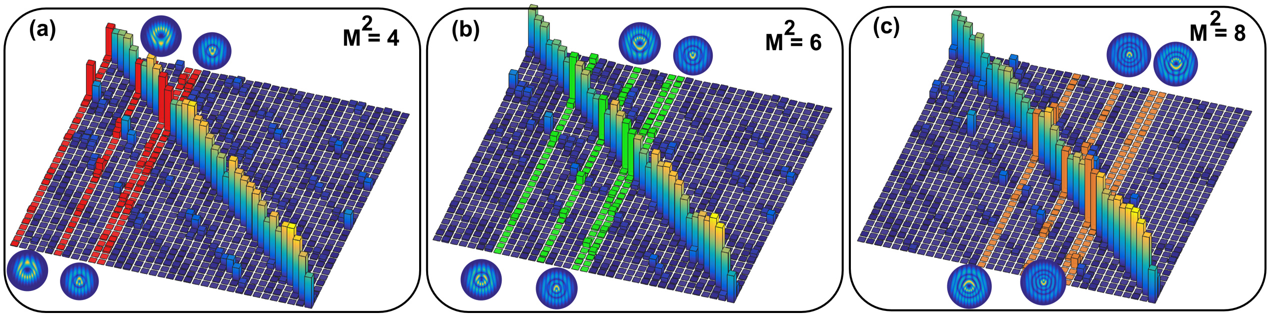

Figure S5. Beam quality factor. Cross-talk table for each wavelength showing the modes that shares the same beam quality factor. (a) λ_1 and $M^2 = 4$, (b) λ_2 and $M^2 = 6$ and (c) λ_3 and $M^2 = 8$.
